# Early anatomical changes and association with photodynamic therapy induced acute exudative maculopathy in patients with macular diseases

**DOI:** 10.1038/s41598-022-13208-y

**Published:** 2022-06-01

**Authors:** Satoshi Honda, Takeya Kohno, Manabu Yamamoto, Kumiko Hirayama, Akika Kyo, Michiko Hirabayashi, Shigeru Honda

**Affiliations:** 1 Department of Ophthalmology and Visual Science, Osaka City University Graduate School of Medicine, Osaka, 545-8585 Japan; 2 Shiraniwa Hospital, Osaka, Japan

**Keywords:** Risk factors, Macular degeneration, Retinal diseases

## Abstract

The purpose of this study was to investigate the occurrence rate and predictors of photodynamic therapy (PDT) induced acute exudative maculopathy (PAEM). This retrospective study included 39 eyes of 39 patients (32 males and 7 females), who were treated with initial PDT. PAEM was defined as an increase in central retinal thickness (CRT) of 15% or more measured by OCT on day 3 after PDT compared with baseline. Sixteen of 39 eyes (41%) were classified in the PAEM+ group. CRT and central choroidal thickness (CCT) were significantly increased at 3 days in the PAEM+ group and significantly decreased at 1 month after PDT in the PAEM- group. In a multiple comparison, neovascular age-related macular degeneration (nAMD) had a significantly higher incidence of PAEM compared to polypoidal choroidal vasculopathy (PCV) and central serous chorioretinopathy (CSC). The incidence of PAEM was lower in PCV and CSC, and higher in nAMD. BCVA at 1 month was significantly worse in the PAEM group, which may be related to visual prognosis after PDT. Since both CRT and CCT decrease at 1 month, the detection of PAEM needs to be assessed a few days after PDT.

## Introduction

Photodynamic therapy (PDT) was developed as a treatment for selective occlusion of choroidal neovascularization (CNV) and has proven to be safe and effective in preventing vision loss in neovascular age-related macular degeneration (nAMD)^1–5^. Since then, PDT has been applied to several macular diseases including polypoidal choroidal vasculopathy (PCV), central serous chorioretinopathy (CSC), myopic choroidal neovascularization, and choroidal hemangioma, and its efficacy has been reported^6–9^. As ocular complications such as choroidal circulatory disturbance, transient vision loss, extensive subretinal hemorrhage and subretinal fibrotic exudative lesions have been reported after PDT with conventional fluence and doses of verteporfin, reduced fluence or dose PDT (RF- or RD-PDT) has also been used as a method to reduce these complications^10–16^.

Although anti-vascular endothelial growth factor (anti-VEGF) therapy has changed the treatment of macular diseases, PDT still plays an important role^17,18^. In addition, in the EVEREST II trial, PDT with anti-VEGF therapy for PCV showed better visual acuity improvement than PDT alone or anti-VEGF therapy alone^6,19^. In chronic CSC, PDT is now also considered to be the first choice over other laser treatments in meta-analyses^7,20–22^. Thus, PDT can improve vision and reduce the mental, physical, and financial burden of patients when used with sufficient consideration of its indications.

Recently, PDT-induced acute exudative maculopathy (PAEM), which develops a few days after PDT, has been reported and has attracted much attention^23–26^. PAEM is thought to be a condition of serous retinal detachment due to an acute fibrinous inflammatory process, which may cause vision loss in the early stage of treatment. The purpose of this study was to examine the incidence of PAEM in macular diseases and to explore the factors associated with it.

## Methods

### Study participants

This was a retrospective, observational case series of 39 eyes of 39 patients with nAMD, PCV and CSC who underwent the initial PDT at the Department of Ophthalmology of Osaka City University Hospital between October 2016 and September 2017. This study was approved by Ethical Committee of Osaka City University Graduate School of Medicine (No. 2019-062), carried out on the basis of the Declaration of Helsinki. Written informed consent was obtained from all patients prior to treatment. The mean age of patients was 72±11 years (range, 43–90 years). Table 1 shows baseline characteristics of the patients. 17 of these 39 eyes (44%) had undergone treatment before PDT, consisting of intravitreal ranibizumab (IVR) in 2 eyes (5%), intravitreal aflibercept (IVA) in 9 eyes (23%), intravitreal bevacizumab (IVB) and IVA in 1 eye (3%), intravitreal IVR and IVA in 3 eyes (8%), vitrectomy due to vitreous hemorrhage in 2 eyes (5%). Time interval from previous treatment to PDT ranged from 2 to 40 months (mean: 5.4 months, median: 2 months).

**Table 1:** Patient characteristics at baseline.

Characteristics	
Number of cases, n	39
Male, n (%)	32 (82)
Age (years), Mean	72 ± 11
Disease subtype	
nAMD	14
PCV	14
CSC	11
Previous treatment, n (%)	17 (44)
Treatment	
Rf-PDT, n (%)	32 (82)
Combination, n (%)	23 (59)
Spot size (µm), Mean	4623 ± 1389
BCVA (logMAR), Mean	0.21 ± 0.33
CRT (µm), Mean	334 ± 151
CCT (µm), Mean	274 ± 92

At the initial visit, all patients had their decimal best-corrected visual acuity (BCVA) measured with a Landolt C chart, had a fundus examination by slit-lamp biomicroscopy, fluorescein and Indocyanine green angiography (FA and IA) and optic coherence tomography using a confocal scanning laser ophthalmoscopy (HRA / Spectralis; Heidelberg Engineering Heidelberg, Germany). We diagnosed nAMD, PCV and CSC using OCT, FA and IA. OCT angiography was also used when the presence of CNV was suspected, which could not be confirmed by these examinations.

### PDT procedure

The PDT protocol was performed by using the full or half-fluence (25 J/cm^2^) for treatment. In cases of nAMD and PCV, full-fluence was used for PDT monotherapy and half-fluence for combined anti-VEGF therapy. Half-fluence PDT was used for CSC. The verteporfin was infused over 10 minutes followed by delivery of an activating light dose of 50 J/cm^2^ from a 689-nm laser system (Carl Zeiss, Dublin, CA) over an 83-second exposure time. The laser spot size for the PDT was the diameter of the region which was determined with FA or IA guided plus a safety zone of 500 µm radius. When anti-VEGF therapy was combined with PDT, it was performed 3 days before PDT.

### Outcome measures

Patients were evaluated by slit-lamp examination, fundus color image and OCT at baseline, 3 days and 1 month after PDT. Though PAEM is defined as a subretinal exudation with or without vision loss occurring within a few days after PDT, there are currently no clear criteria^23,24,26^. We have clearly defined PAEM to allow detection of minor changes after PDT: an increase in central retinal thickness (CRT) of 15% or more measured by OCT on day 3 after PDT compared with baseline. Patients were divided into PAEM+ and PAEM− groups according to this criterion. The bacillary detachment (BALAD) was evaluated after PDT in PAEM+ group. BCVA was also measured at baseline and 1 month after PDT. The BCVA was converted to logarithm of the minimum angle of resolution (logMAR) units before analysis. Age, gender, type of disease (nAMD, PCV or CSC), PDT irradiation method (full- or half-fluence), presence of combined therapy, history of treatment before PDT, baseline BCVA, CRT, and central choroidal thickness (CCT) were selected for factors associated with the development of PAEM.

### Statistical analysis

Changes in CRT, CCT BCVA (logMAR) from baseline were assessed using the Wilcoxon signed rank test with Bonferroni correction. In order to assess the associations between the incidence of PAEM after PDT and the various parameters, we performed univariate analyses using the chi-square test with categorial data and the Mann–Whitney U test with the parametric data. IBM SPSS Statistics 24.0 (IBM Japan, Ltd., Tokyo, Japan) was used for statistical analysis, in which *p* values < 0.05 were regarded as significant.

## Results

**Figure 1:**
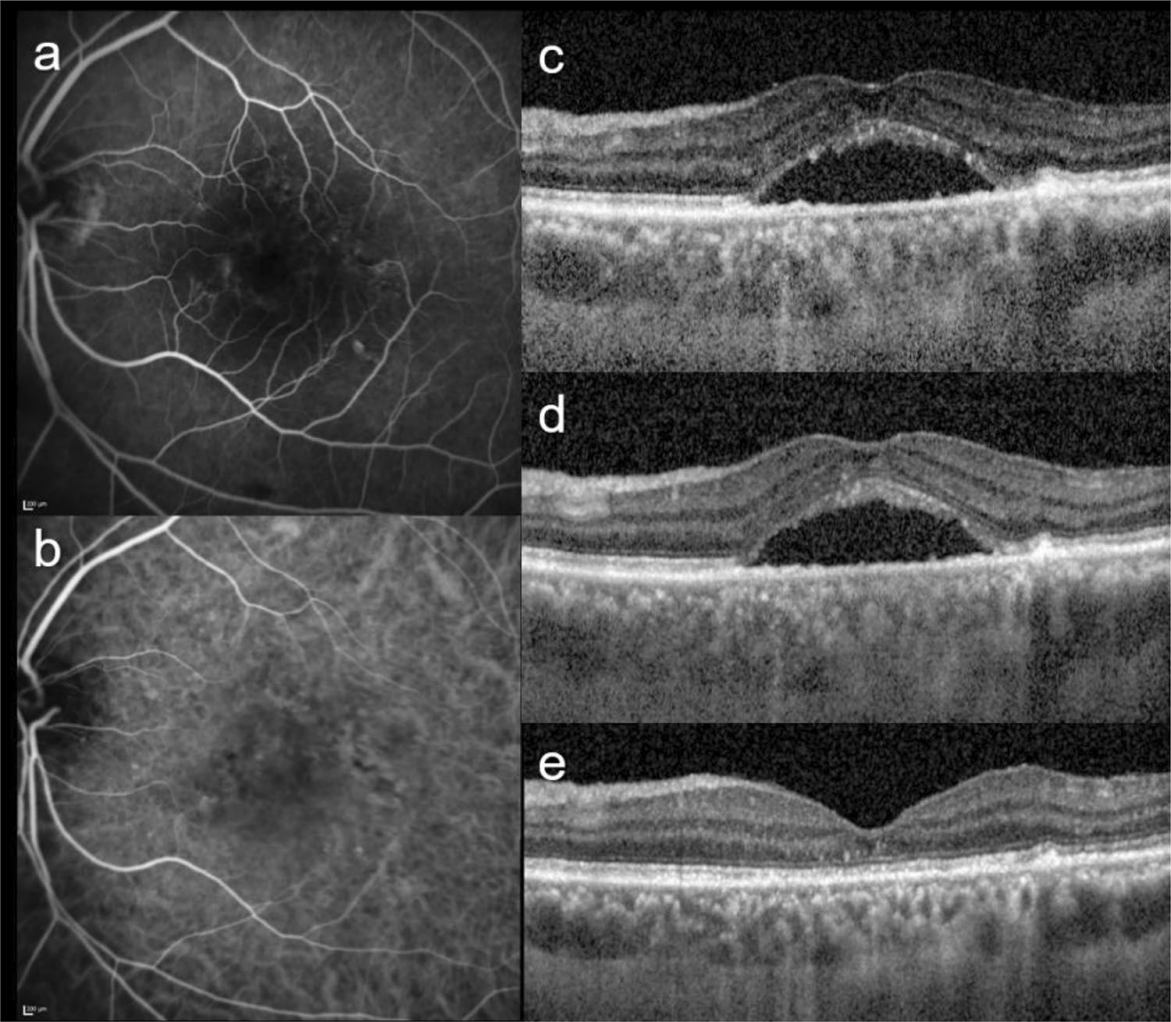
This is a representative case of PAEM-. Fluorescein angiography (**a**), indocyanine green angiography (**b**), and horizontal line of optic coherence tomography images of patients at baseline (**c**), 3 days follow-up (**d**) and 1 month follow-up (**e**) after photodynamic therapy (PDT). 60-year-old-male with central serous chorioretinopathy in the left eye. Central retinal thickness was 358 µm at baseline, 364 µm (+ 1.7%) at 3 days, 122 µm (− 65.9%) at 1 month after PDT. Central choroidal thickness was 338 µm at baseline, 341 µm (+ 0.9%) at 3 days, 255 µm (− 24.6%) at 1 month after PDT. Complete resolution of sub retinal fluid was seen at 1 month follow-up.

**Figure 2:**
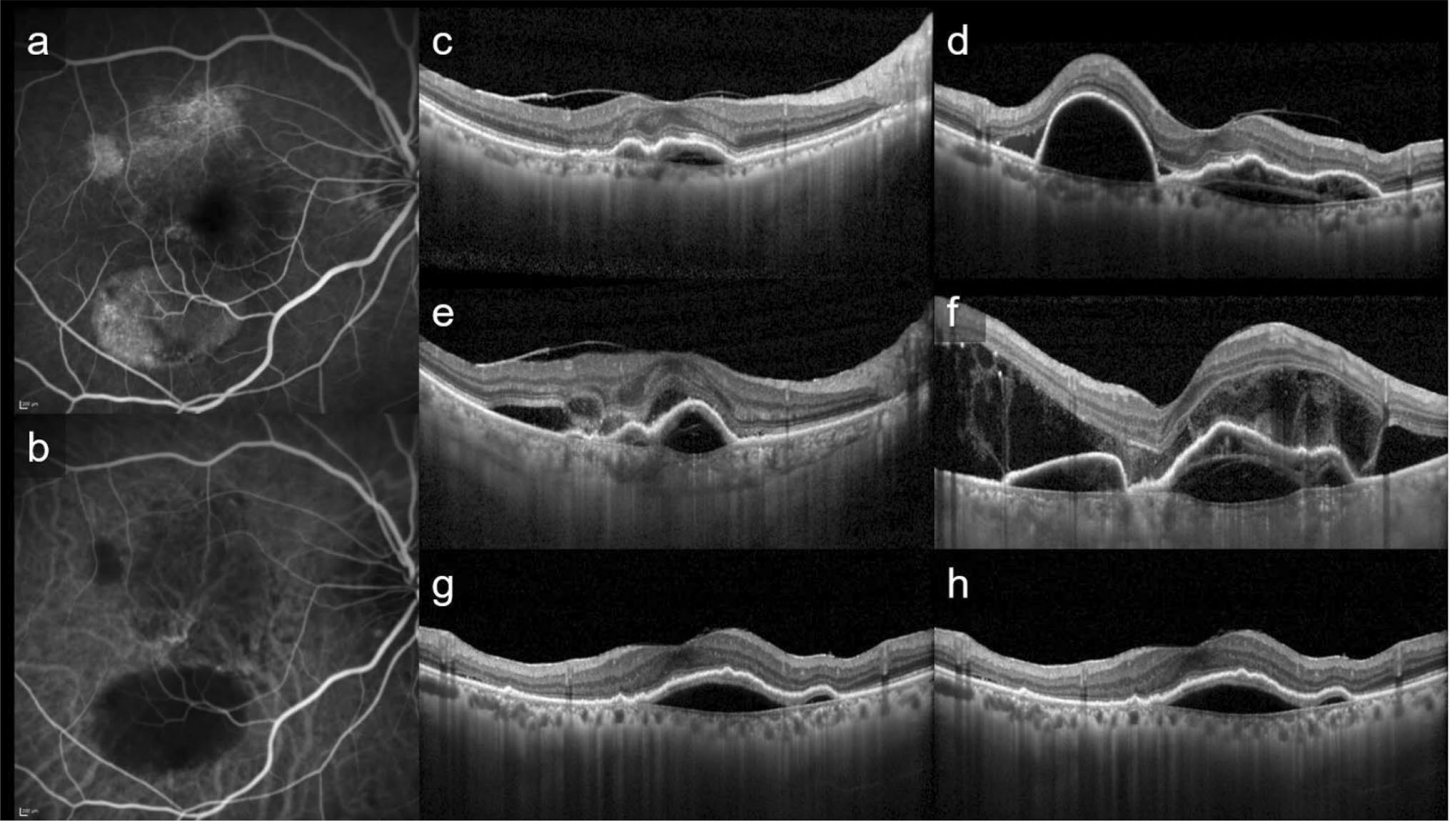
This is a representative case of PAEM+. Fluorescein angiography (**a**), indocyanine green angiography (**b**), and horizontal (**c**, **e**, **g**) and vertical (**d**, **f**, **h**) line of optic coherence tomography images of patients at baseline (**c**, **d**), 3 days follow-up (**e**, **f**) and 1 month follow-up (**g**, **h**) after photodynamic therapy (PDT). 82-year-old-female with polypoidal choroidal vasculopathy in the light eye. Central retinal thickness was 361 µm at baseline, 538 µm (+ 49.0%) at 3 days, 334 µm (− 7.5%) at 1 month after PDT. Central choroidal thickness was 135 µm at baseline, 270 µm (+ 100.0%) at 3 days, 126 µm (− 6.7%) at 1 month after PDT. The subretinal fluid increased at 3 days follow-up, with complete resolution at 1 month follow-up. The bacillary detachment was also seen only at 3 days follow-up.

Typical two cases treated with PDT are shown in Figs. 1 and 2. The subjects analyzed in this study included 39 eyes of 39 patients (32 eyes of 32 men; 7 eyes of 7 women) with nAMD, PCV and CSC. Table 1 shows baseline characteristics of the patients in this study. Mean patient age was 72 ± 11 years. nAMD, PCV, and CSC were included in 14, 14, and 11 eyes, respectively, of the total cases. Seventeen eyes had a history of treatment: 5 eyes were treated with ranibizumab, 14 eyes with aflibercept, 1 eye with bevacizumab, and 2 eyes with vitrectomy. Half-fluence PDT was performed in 32 eyes, and the combination with anti-VEGF agent was performed in 23 eyes. There was no additional treatment with or without PAEM by 1 month after PDT.

**Figure 3:**
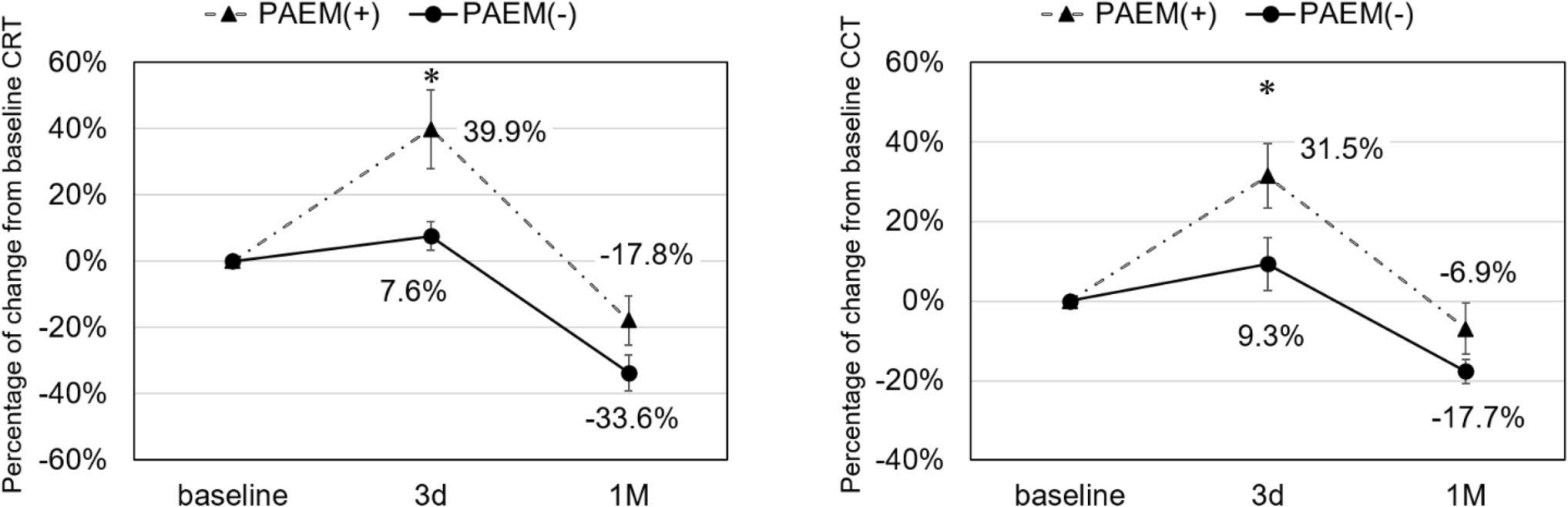
Change in central retinal thickness (CRT) (**a**) and central choroidal thickness (CCT) (**b**) from baseline. In the comparison between the two groups, the rate of change in CRT and CCT was significantly increased in the PAEM group at 3 days after PDT, with no significant difference at 1 month (CRT and CCT: 3 days, *p* < 0.05 and *p* < 0.05; 1 month, *p* = 0.17 and *p* = 0.21).

Sixteen of 39 eyes (41%) were classified in the PAEM+ group. Table 2 shows changes in BCVA, CRT and CCT. In PAEM+ group, mean BCVA was 0.27 ± 0.08 before PDT and 0.35 ± 0.09 after 1 month, showing a significant worsening (*p* < 0.05). In the PAEM- group, mean BCVA was 0.17 ± 0.07 at baseline and 0.20 ± 0.07 at 1 month, showing no significant difference (*p* = 0.29). In the PAEM+ group, there was a significant increase in CRT and CCT at 3 days compared to baseline, with no significant difference at 1 month (3 days, *p* < 0.05 and <0.001; 1 month, *p* = 0.26 and 0.48). In the PAEM- group, there was no significant change in CRT and CCT at 3 days, with significant decrease at 1 month (3 days, *p* = 0.17 and 1.00; 1 month, *p* < 0.001 and < 0.001). In the comparison between the two groups, the rate of change in CRT and CCT was significantly increased in the PAEM group at 3 days after PDT, with no significant difference at 1 month (CRT and CCT: 3 days, *p* < 0.05 and *p* < 0.05; 1 month, *p *= 0.17 and *p* = 0.21) (Fig. 3). The BALAD was observed in 11 out of 16 eyes (69%) in PAEM+ group, 8 out of 11 in nAMD, 3 out of 3 in PCV, 0 out of 2 in CSC. In univariate analysis comparing the two groups, there were significant differences in age and clinical diagnosis as baseline factors (Age: *p* < 0.05, Clinical diagnosis: *p* < 0.05) (Table 3).

**Table 2:** Visual and anatomical changes at 3 days and 1 month after PDT compared to baseline.

	Baseline	3d	*p* Value	1 M	*p* Value
BCVA (logMAR)					
PAEM+	0.27 ± 0.08			0.35 ± 0.09	** < 0.05**
PAEM−	0.17 ± 0.07			0.20 ± 0.07	0.29
CRT (µm)					
PAEM+	347 ± 39	483 ± 61	** < 0.05**	285 ± 30	0.26
PAEM−	325 ± 33	345 ± 35	0.17	196 ± 17	** < 0.001**
CCT (µm)					
PAEM+	244 ± 22	309 ± 22	** < 0.001**	227 ± 23	0.48
PAEM−	294 ± 19	303 ± 15	1.00	242 ± 18	** < 0.001**

Significant values are in bold.

**Table 3:** Univariate analysis of factors associated with PAEM.

Characteristics	PAEM(+)	PAEM(−)	*p* Value
Number, n	16	23	
Male, n (%)	13 (81)	19 (83)	0.617
Age (years), mean	76	69	** < 0.05**
Clinical diagnosis, n (nAMD : PCV : CSC)	11 : 3 : 2	3 : 11 : 9	** < 0.05**
Previous treatment, n (%)	9 (56)	8 (35)	0.158
Rf-PDT, n (%)	12 (75)	20 (87)	0.294
Combination, n (%)	10 (63)	13 (57)	0.485
Spot size (µm), mean	4763 ± 1285	4526 ± 1478	0.608
BCVA (logMAR), mean	0.09 ± 0.14	0.03 ± 0.12	0.172
CRT (µm), mean	347 ± 146	325 ± 156	0.649
CCT (µm), mean	244 ± 87	294 ± 92	0.100

Significant values are in bold.

**Figure 4:**
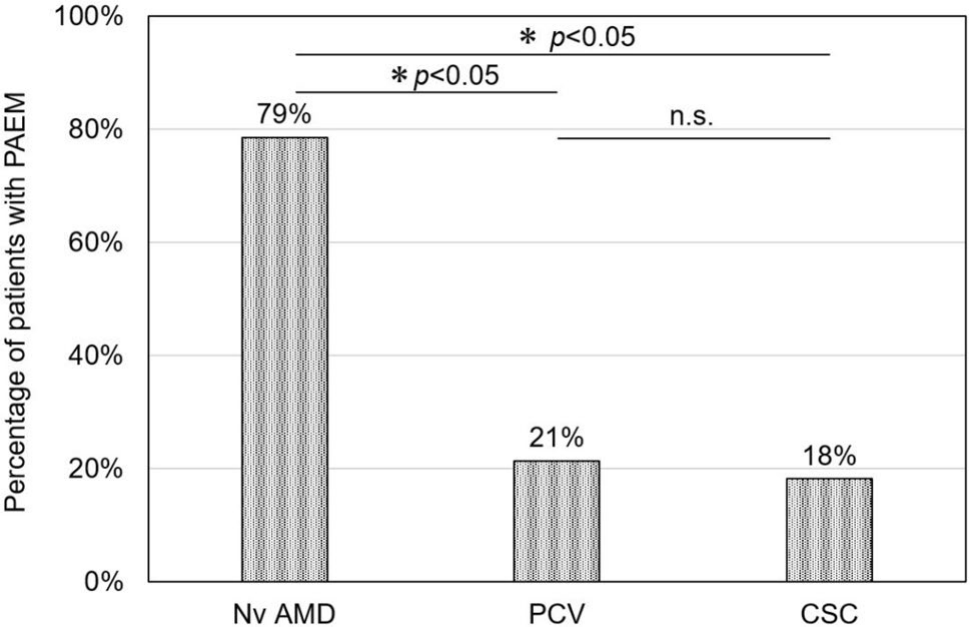
Incidence of PAEM by disease subtype. The incidence of PAEM was 79% in nAMD, 21% in PCV, and 18% in CSC. In a multiple comparison of the three groups, nAMD had a significantly higher incidence of PAEM compared to PCV and CSC (nAMD versus PCV: *p* < 0.05, nAMD versus CSC: *p* < 0.05, PCV versus CSC: *p* = 1.00).

The results of comparison between the three groups according to clinical diagnosis showed significant differences in the incidence of PAEM and age (incidence of PAEM: *p* < 0.05, age: *p* < 0.001) (Table 4). In a multiple comparison of the three groups, nAMD had a significantly higher incidence of PAEM compared to PCV and CSC (nAMD versus PCV: *p* < 0.05, nAMD versus CSC: *p* < 0.05, PCV versus CSC: *p* = 1.00) (Fig 4).

**Table 4:** Comparison of rate of PAEM and age between disease subtype.

Characteristics	nAMD	PCV	CSC	*p* value
Number of cases, n	14	14	11	
Number of PAEM, n (%)	11 (79)	3 (21)	2 (18)	** < 0.05**
Age (years), mean	79 ± 6	74 ± 6	60 ± 11	** < 0.001**

Significant values are in bold.

## Discussion

In the present study, 16 of 39 eyes (41%) were defined as PAEM, and the incidence of each disease was 79% for nAMD, 21% for PCV, and 18% for CSC. In their study, Mammo and Forooghian^23^ reported that in 47 CSC patients treated with PDT in 52 eyes and AMD in 5 patients treated with PDT in 6 eyes, PAEM developed in 1.4% (CSC: 1 eye, AMD: 1 eye) of the patients. However, only patients who complained of decreased visual acuity a few days after the procedure were added to the examination, so it is possible that mild asymptomatic cases were missed. Manayath et al.^24^ reported on 177 patients with 155 eyes (84 sessions for CSC, 66 sessions for PCV, 5 sessions for PCV with CSC-like leakage, 15 sessions for occult CNV, and 7 sessions for posterior tumors). A total of 8 patients (4.52%) had PAEM: 6 with PCV, 1 with occult CNV, and 1 with CSC. This report also suggests that PAEM without visual impairment may have been overlooked. The incidence by disease was PCV (8.45%), occult CNV (6.67%), and CSC (1.19%), respectively. In this study, the definition of the onset of PAEM was strictly defined as a 15% increase in CRT early after PDT, which resulted in a more sensitive detection of PAEM, and the overall rate was higher in all diseases than previously reported. Our study also resulted that BCVA had decreased in the PAEM+ group 1 month after PDT, but the long-term effect has been still unknown. Fernandez-Vigo et al. reported that PAEM did not affect the mean BCVA gain at 3 months after PDT in chronic CSC, so further investigation is warranted^26^.

Some reports have shown that PAEM is more likely to occur in CNV, followed by PCV, and rarely in chronic CSC^24,27,28^. Recently, Fernandez-Vigo et al.^26^ reported that in 92 eyes with CSC PAEM was observed 28 eyes (30.4%) with the criteria that define PAEM as an increase of 50 µm or more in the SRF height 3 days after PDT. While this percentage seemed to be higher than in other previous studies, including ours as CSC, that study was included the patients with CNV, which may be the reason for the higher percentage despite in CSC. According to Holtz et al. occult CNV and classic CNV express verteporfin-targeted low-density lipoprotein receptors, which makes the RPE vulnerable to oxidative stress^29^. In addition, PAEM may occur in CSC because of the large choroidal vessels and the lack of components which mask RPE, but the incidence may be lower because of the absence of neovascularization^30^. These hypotheses are also supported by the higher incidence of PAEM in nAMD compared to PCV and CSC in this study. In addition, according to previous reports, CNV is the most likely disease to develop PAEM, followed by PCV, and it is rare in CSC. In the present study, nAMD had a higher incidence, but PCV and CSC had a lower incidence, which may be consistent with previous reports.

Although there is no clear pathogenesis for PAEM, several factors have been suggested. Schmidt et al. stated that the choroidal capillary occlusion effect of PDT causes oxidative stress in vascular endothelial cells, which stimulates histamine production and dissociation of intercellular tight junctions, resulting in increased permeability of choroidal vessels, leading to exudative changes and edema^3^. It has also been suggested that occlusion of the choriocapillaris after PDT causes choroidal ischemia and inflammatory changes, resulting in the production of VEGF and other substances and exudative changes in the retina and choroid^31^. In addition, BALAD was observed in 11 out of 16 eyes in PAEM+ group. It was reported as a new OCT finding, the main mechanism of which was considered to be exudative retinal detachment due to the disruption of outer blood-retina barrier^32^. PDT may cause RPE pump dysfunction which can further exacerbate fluid accumulation^33^. The BALAD could also be considered evidence that PAEM is caused by RPE dysfunction. Interestingly, in the present study, there was a significant increase in CCT as well as CRT 3 days after PDT in the PAEM+ group, but not in the PAEM- group. A recent report using OCT angiography showed vascular occlusion of the choriocapillaris corresponding to the treated area at 3 days after PDT and recanalization of the choroidal capillary plate at 6 days^34^. The fact that temporary occlusion of choriocapillaris occur early after PDT may be considered supportive of our results.

There have been reported that risk factors for the development of PAEM include age 65 years or older, PCV, BCVA 20/40 or better, PDT spot size of 3500 µm or larger, reduced fluence PDT, and initial PDT^24^. In the current study, nAMD and older age were associated with the incidence of PAEM, and there was no difference in BCVA or the method of PDT such as reduced fluence, spot size or combination therapy. PAEM was seen in 14 of 32 eyes (44%) with PDT spot size of 3500 µm or larger and in 2 of 7 eyes (29%) under 3500 µm. The incidence of PAEM appeared to be lower in the group with spot size under 3500 µm, possibly because 4 of the 7 eyes were CSC cases. The resolution of PAEM was observed in all cases at 1month after PDT, although no additional treatment was administered at the time of PAEM onset. To date, there is no consistent opinion on the treatment of PAEM; thus, a variety of treatments have been reported, including anti-VEGF therapy and steroid therapy (systemic or local)^23–25,31^. This may be a point that cannot be concluded from this study due to the small sample size and differences in the selection criteria of combination therapy among disease subtypes.

In conclusion, PAEM was clearly and rigorously defined in this study, and the incidence of PAEM was lower in PCV and CSC, and higher in nAMD. BCVA at 1 month was significantly worse compared with baseline in the PAEM group, which may be related to visual prognosis after PDT. Since both of CRT and CCT decrease at 1 month after PDT, the detection of PAEM needs to be assessed a few days post-PDT. This study was limited by a small sample size, a non-randomized and retrospective study design and a short follow-up period. Further prospective studies with a larger number of patients will be required to confirm the association factors with PAEM found in the present study.

## Supplementary Information

Supplementary Information 1.

## Data Availability

All data generated or analysed during this study are included in this published article and its supplementary information files.
